# Supplementary material for: Antibacterial potential of Luidia clathrata (sea star) tissue extracts against selected pathogenic bacteria
Source: PLoS One. 2023 Mar 2;18(3):e0281889. doi: 10.1371/journal.pone.0281889 (PMC9980771; doi:10.1371/journal.pone.0281889)
Supplement: Attachment — Submitted filename: Parajuli_PONE-D-22-30432R1_Response.docx [file pone.0281889.s002.docx]

**PONE-D-22-30432**

**Antibacterial Potential of *Luidia clathrata* (Sea Star) Tissue Extracts Against Selected Pathogenic Bacteria**

**PLOS ONE**

1. **Response to Reviewer 1**

We would like to thank the reviewer for the valuable comments/suggestions. We summarize our responses as follows.

Q1: Writing issues and grammatical errors

Thank you for identifying these issues. We have fixed these issues (formatting, typos and grammatical errors) in the updated draft.

Q2: Clarification on novelty of the research

Thank you for your comment. To the best of our knowledge, this is the first scientific work explaining the antimicrobial potential of different body tissues of *Luidia clathrata* against ten different pathogenic bacteria. Specifically, our effort to explore the therapeutic potential of gonads from the *L. clathrata* is unique. By sharing our results with the scientific community, we will be contributing to the advancement of this field and helping to spur future research efforts aimed at identifying the active compounds responsible for the observed antibacterial activity. We believe, our research is novel.

2. **Response to Reviewer 2**

We would like to thank the reviewer for the valuable comments/suggestions. We summarize our responses as follows.

Q1: Abstract revision (Which pathogens were used in the experiment? Provide detailed names of the pathogens. Mention the tissues used in the experiment.)

We have done a major revision of the abstract in light of reviewer’s comments. Please refer to the highlighted lines throughout the abstract.

Regarding the bacterial names, we have mentioned the bacterial species and strain in detail in the methodology section. However, as the reviewer wanted us to add the names in the abstract, we have now incorporated them.

We also have specified tissues/organs used in the experiment- in the abstract now.

Q2: Clarification on Material and Methods

Thank you for your questions and pointing out deficiencies. We have incorporated all the issues, as suggested.

Briefly,

- We have added the sample size
- We have mentioned the acclimation period
- We have added the tissue/organs used in the experiment
- Regarding the purity and concentration of the extractants used, we have updated the information
- We have updated the detailed information about the instruments used.

In addition, we like to inform you that the decantation period was 5 minutes and the pressure for rotary evaporator was 713 mmHg at 40-45^o^C (Reference 1).

Q3: Clarification on the chemical analysis of the extracts used

Thank you for your suggestion. This result is the first part of our experiment that we would like to share. We will follow up your suggestion on chemical analysis of the extracts used in our follow up research with standard methodology.

**References:**

1. Souza, C. R. F., Schiavetto, I. A., Thomazini, F. C., & Oliveira, W. P. D. (2008). Processing of Rosmarinus officinalis Linne extract on spray and spouted bed dryers. *Brazilian Journal of Chemical Engineering*, *25*, 59-69. <https://doi.org/10.1590/S0104-66322008000100008>
